# Transforming Translation: Impact of *Clinical and Translational Science*

**DOI:** 10.1111/cts.12380

**Published:** 2016-01-15

**Authors:** JA Wagner, DL Kroetz

**Affiliations:** ^1^ Takeda Pharmaceuticals International CoCambridgeMassachusettsUSA; ^2^ Department of Bioengineering and Therapeutic SciencesUniversity of CaliforniaSan FranciscoCaliforniaUSA

What is translational medicine? This question has been vigorously debated by various constituencies for many years. One recent, encompassing definition of translational medicine was developed for the Strategic Plan in service of the American Society for Clinical Pharmacology and Therapeutics (ASCPT).^1^, ^2^ Not coincidentally, the ASCPT Strategic Plan was aptly entitled “Transforming Translation.”

“Implementation of the ASCPT Strategic Plan will be guided by a broad and inclusive description of translational medicine to reflect the diversity of scientific disciplines involved in translational research within our Society. For the purpose of this document, translational research, translational science, and translational medicine will be used interchangeably with a unifying principle that the ultimate purpose is to improve human health via a “bench to bedside” approach. There are many definitions of translational medicine as well as translational science and translational research, which provide context for ASCPT's efforts. John Hutton^3^ defines translational research as “Research [that] transforms scientific discoveries arising from laboratory, clinical or population studies into new clinical tools and applications that improve human health by reducing disease incidence, morbidity, and mortality.” Another perspective^4^ is “Translational research fosters the multidirectional integration of basic research, patient‐oriented research, and population‐based research, with the long‐term aim of improving the health of the public.”

From ASCPT's perspective, translational medicine is a multifaceted discipline with a focus on translational therapeutics. In a broad sense, translational medicine bridges across the discovery, development, regulation, and utilization spectrum. It may include application of research findings from genes, proteins, cells, tissues, organs, and animals, to clinical research in patient populations, all aimed at optimizing and predicting outcomes in specific patients. For clinical pharmacology, the focus of translational research is on the discovery, development, regulation, and use of pharmacologic agents to improve clinical outcome and inform optimal use of therapeutics in patients. In addition, translational research in clinical pharmacology may include evaluation of various biomarkers of pharmacologic response and assessing the linkage between biomarker response and clinical endpoints in patients. Our broad description also includes how the response to a therapeutic intervention in a particular disease may translate to a response in another disease, as well as translation of safety signals across species and/or patient populations. Translational research is bolstered by quantitative, model‐based, and mechanistic understanding of disease biology and pharmacology. Consequently, core disciplines, including clinical pharmacology, pharmacogenomics, systems pharmacology, precision medicine, as well as others play an integral role in enabling translational research and translational medicine.”^1^

Published research on translational medicine, translational science, and translational research has exploded, with over 7,000 PubMed citations in the last year alone (**Figure** 1), and yet the cross‐therapeutic needs of the translational medicine community remain underserved. As described by Rocci^2^ in this issue, one important intent of the ASCPT Strategic Plan is to “transform translation” and focus the attention of the Society on translational research. Thus, a major aim of *Clinical and Translational Science* (*CTS*), now under the auspices of ASCPT, is to be a beacon and organizing principle for the field of translational medicine. The broad definition developed by ASCPT for translational medicine serves as a goal post for the focus of *CTS*. With this definition in mind, the journal highlights original research that helps bridge laboratory discovery with the diagnosis and treatment of human disease. Publications may appear as full Articles, Commentaries, Phase Forward (well‐conducted, concisely reported, and relevant clinical trials), Reviews, or Tutorials. *CTS* also includes invited didactic content that covers the connections between clinical pharmacology and translational medicine. These additional features provide context for research articles and facilitate understanding for a wide array of individuals interested in clinical and translational science. *CTS* welcomes high quality, scientifically sound original manuscripts focused on clinical pharmacology and translational science, including animal, *in vitro*, *in silico*, and clinical studies supporting the breadth of drug discovery, development, regulation, and clinical use of drugs. Example topics of interest are displayed in **Table** 1.

**Figure 1:**
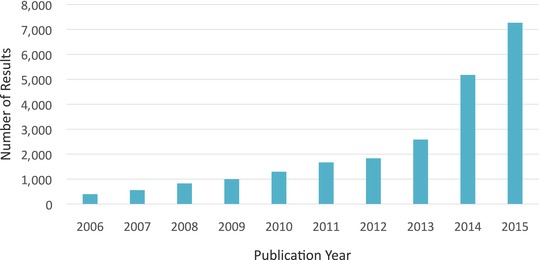
Translational medicine, translational science, and translational research publishing has grown dramatically over the last decade. Increase in publications identified by the key words “translational medicine” [or] “translational science” [or] “translational research” in PubMed in 2006 through 2015 and plotted by year.

**Table 1:** Example topics of interest for *Clinical and Translational Science*

Translational medicine, including studies focused on interrogation/evaluation of mechanism‐of‐action, human physiology, and interruption of disease pathophysiology
Hypothesis generating nonclinical and clinical studies, including small clinical trials
Models of human disease and their therapeutic implications
Studies that guide Phase 2 dose selection
Studies that identify or support biomarkers that can be used at any stage of drug development
Studies that demonstrate effective communication between basic and clinical science
Regulatory and public health policy implications of translational studies
Pharmacokinetic/pharmacodynamic (PK/PD) and quantitative pharmacology as these relate to translational medicine
Precision medicine

Biomarkers have an enormously important role in the definition of translational medicine and serve as the common language of translational sciences. A biomarker has been defined as “a characteristic that is objectively measured and evaluated as an indicator of normal biologic processes, pathogenic process, or pharmacologic responses to a therapeutic intervention.”^5^ This comprehensive definition of biomarkers arose from the April 1999 US Food and Drug Administration (FDA)/National Institutes of Health (NIH) consensus conference on “Biomarkers and Surrogate Endpoints: Advancing Clinical Research and Applications,” and emphasized that biomarkers are medical measurements, including physiological measurements, blood tests, molecular analyses of biopsies, genetic or metabolic data, and measurements from images. Biomarkers serve as the “glue” that adheres many of the component translational disciplines together, and are undergoing a renaissance of increased interest. In this issue Li *et al*.^6^ discuss the innovative use of imaging as a biomarker in oncology. The uptick in interest in the field of biomarkers include activities such as US Congressional scrutiny (in the form of the 21st Century Cures legislation), the FDA's biomarker qualification pathway, the FDA/NIH biomarker taxonomy effort, the Foundation for the National Institutes of Health (FNIH) Biomarkers Consortium, Brookings / FDA, efforts by Centers of Excellence in Regulatory Science and Innovation, Critical Path Institute (C‐PATH), National Biomarkers Development Alliance, and the Institute of Medicine (IOM) report on biomarkers and surrogate endpoints. The high level of community attention underlines the importance of biomarkers in translational medicine, and the corresponding role biomarkers are expected to have in *CTS*.

Another tenet of the ASCPT definition of translational medicine is that translational research is bolstered by quantitative, model‐based, and mechanistic approaches. Sheiner's learn‐confirm paradigm^7^ leads naturally to the idea of translational medicine in which pharmacometric models of drug efficacy and safety link preclinical and clinical data, particularly biomarker information, with prior knowledge of disease, and provide a quantitative approach to improving drug development and decision‐making, as well as translational medicine more generally. Pharmacometric models contribute to successful and efficient drug development by illuminating drivers of efficacy and safety outcomes, by predicting the outcome of future events using simulations of alternative study designs and understudied patient populations, and by estimating the probability of a successful trial given a set of well‐articulated assumptions. These concepts culminate in the idea of model‐based drug development, wherein the sciences of pharmacokinetics, pharmacodynamics, statistics, clinical pharmacology, and therapeutic areas are integrated to drive more robust decision‐making. Key benefits of this approach include quantitative integration of translational medicine knowledge and discussion of important assumptions.

Pharmacogenomics is often considered the poster child of translational medicine. Defined by the International Conference of Harmonization as the “study of variations in DNA sequences as related to drug response,” pharmacogenomics has long been recognized for its contribution to interindividual variability in drug response and toxicity, and an increasing number of drug labels include recommendations for genotype‐guided dosing of therapeutics.^8^, ^9^ In recent years, the focus of pharmacogenomics research has evolved from the traditional emphasis on discovering genetic determinants of drug exposure (pharmacokinetics), to a broader definition that includes genes and pathways that underlie the pharmacologic and toxic response to therapeutics (pharmacodynamics). In this issue, Khalil *et al*.^10^ and Hamadeh *et al*.^11^ identify the impact of pharmacogenomics in new populations. There remains a significant need for continued efforts to implement current pharmacogenomics knowledge into patient care. In addition, unbiased approaches using genome‐wide genotyping and sequencing are being implemented to identify novel genes that contribute to therapeutic efficacy and toxicity. Pharmacogenomic research holds the promise of novel drug design targeting specific DNA sequences and a molecular understanding of drug response and toxicity that can be incorporated into drug development and therapeutic utilization. Translational medicine requires a systems level understanding of variability in drug response that considers genetic variation in the context of environmental and other nongenomic influences. *CTS* welcomes pharmacogenomics contributions that span the bench‐to‐bedside spectrum of translational sciences.

One of the most innovative approaches to translational medicine evolves the classic “bench‐to‐beside” approach to “bedside‐to‐bench‐to‐bedside,”^12^ and, thus, integrates the many facets of translational medicine. One recent, powerful example highlights the creative energy, innovation, interplay of cutting‐edge technology, and benefit to patients of this approach. In 2003, a mutation in proprotein convertase subtilisin/kexin type 9 (PCSK9) was discovered and associated with remarkably low cholesterol. This clinical observation drove interest in target validation that elucidated a key role for PCSK9 binding low‐density lipoprotein (LDL) receptors, which in turn control LDL cholesterol levels in the circulation. Human genetics prompted confirmation that PCSK9 null mice also had remarkably low cholesterol. These bedside‐to‐bench findings drove the search and discovery of antibody inhibitors of PCSK9, which could reduce degradation of LDL receptors resulting in lower cholesterol levels. Twelve years after the initial bedside observations, two monoclonal antibodies, alirocumab and evolocumab, have been shown to dramatically reduce LDL cholesterol in patients with hypercholesterolemia.^13^ Although statins have been the mainstay of cholesterol‐reducing therapy for decades, PCSK9 monoclonal antibodies will now have an important role in statin‐intolerant and unresponsive patients, serving this unmet medical need.

In addition to the important scientific components that make up the field of translational medicine, there are societal trends, including “democratization” of science and health care, the explosion of social media in science and health care, the increasing role of the patient, precompetitive collaboration, and the globalization of research. Exciting developments in translational medicine are occurring outside the United States, which necessities a broad, international approach including Europe, United States, Asia, and emerging economies. For example, China has seen double‐digit growth in its biotechnology industry and has gone from being one of the slowest to one of the fastest nations in the adoption of new biotechnologies. *CTS* intends to embrace these societal trends as the field of translational medicine evolves.

Translational sciences research holds the promise of more effective and safe therapeutics. Inherent in this promise is the need for robust bench‐to‐bedside and bedside‐to‐bench‐to‐bedside research that embraces the tremendous technological advancements and advanced analysis methods that are driving modern data‐driven discoveries. Our vision for *CTS* is that it will provide a widely recognized platform for the broad dissemination of high quality translational science research that will lead to the optimal use of therapeutics. We encourage submissions from the diverse fields that span the translational sciences spectrum across diverse therapeutic areas and across the breadth of discovery, development, regulation, and clinical use of therapeutics.

## Conflict of Interest/Disclosure

J.A.W. is an employee of Takeda Pharmaceutical International Co. and may potentially own stock and/or hold stock options in the Company. D.L.K. has no conflicts to disclose.

## References

[1] American Society for Clinical Pharmacology and Therapeutics. ASCPT Strategic Plan: Transforming Translation http://www.ascpt.org/portals/8/strategicplan/index.html (2015).**

[2] Rocci, M.L. Jr. The American Society for Clinical Pharmacology and Therapeutics (ASCPT) – a rich heritage en route to an exciting future. Clin. Transl. Sci. 9: 6–8 (2016). 26678381 10.1111/cts.12381 PMC5351324

[3] Wang, X. A new vision of definition, commentary, and understanding in clinical and translational medicine. Clin. Transl. Med. 1: 5 (2012). 23369396 10.1186/2001-1326-1-5 PMC3552566

[4] Rubio, D.M. *et al* Defining translational research: implications for training. Acad. Med. 85: 470–475 (2010). 20182120 10.1097/ACM.0b013e3181ccd618 PMC2829707

[5] NIH Definitions Working Group . Biomarkers and surrogate endpoints in clinical research: definitions and conceptual model In Biomarkers and Surrogate Endpoints: Clinical Research and Applications (ed. Downing G.L.) 1–9 (Elsevier, Amsterdam, 2000).

[6] Li, C.H. *et al.* Comparative effects of CT imaging measurement on RECIST endpoints and tumor growth kinetics modelling. Clin. Transl. Sci. 9: 43–50 (2016). 26790562 10.1111/cts.12384 PMC4760886

[7] Sheiner, L.B. Learning versus confirming in clinical drug development. Clin. Pharmacol. Ther. 61: 275–291 (1997). 9084453 10.1016/S0009-9236(97)90160-0

[8] Ehmann, F. , Caneva, L. & Papaluca M. European Medicines Agency initiatives and perspectives on pharmacogenomics. Br. J. Clin. Pharmacol. 77: 612–617 (2014). 24433361 10.1111/bcp.12319 PMC3971978

[9] Yip, V.L. , Hawcutt, D.B. & Pirmohamed, M. Pharmacogenetic Markers of Drug Efficacy and Toxicity. Clin. Pharmacol. Ther. 9861–9870 (2015). 10.1002/cpt.135 25870137

[10] Khalil, B.M. *et al.* Genetic and nongenetic factors affecting clopidogrel response in the egyptian population. Clin. Transl. Sci. 9: 23–28 (2016). 26757134 10.1111/cts.12383 PMC4760893

[11] Hamadeh, I.S. *et al.* Impact of *GGCX, STX1B* and *FPGS* polymorphisms on warfarin dose requirements in European Americans and Egyptians. Clin. Transl. Sci. 9: 36–42 (2016). 26751406 10.1111/cts.12385 PMC4760888

[12] Recke, A. & Ludwig, R.J. From bedside to bench–reverse translational medicine. Scientific lessons from revertant mosaicism in ‘knockout’ humans. Exp. Dermatol. 23(8): 549–550 (2014). 24961831 10.1111/exd.12475

[13] Everett, B.M. , Smith, R.J. & Hiatt, W.R. Reducing LDL with PCSK9 inhibitors–the clinical benefit of lipid drugs. N. Engl. J. Med. 373: 1588–1591 (2015). 26444323 10.1056/NEJMp1508120

